# Refining the hospitalization rate: A mixed methods approach to differentiate primary COVID-19 from incidental cases

**DOI:** 10.1016/j.infpip.2024.100371

**Published:** 2024-05-15

**Authors:** M. Misailovski, D. Koller, S. Blaschke, M. Berens, A.M. Köster, R. Strobl, R. Berner, P. Boor, M. Eisenmann, S. von Stillfried, D. Krefting, M. Krone, J. Liese, P. Meybohm, G. Ulrich- Merzenich, S. Zenker, S. Scheithauer, E. Grill

**Affiliations:** a Department of Infectious Control and Infectious Diseases, University Medical Center Göttingen, Göttingen, Germany; b Institute of Medical Data Processing, Biometrics and Epidemiology, Faculty of Medicine, LMU Munich, Munich, Germany; c Emergency Department, University Medical Center Göttingen, 37075 Göttingen, Germany; d German Center for Vertigo and Balance Disorders, Faculty of Medicine, University Hospital, LMU Munich, Munich, Germany; e Department of Pediatrics, University Hospital and Medical Faculty Carl Gustav Carus, TUD Dresden University of Technology, Dresden, Germany; f Institute of Pathology, RWTH Aachen University Hospital, Aachen, Germany; g Infection Control and Antimicrobial Stewardship Unit, University Hospital Wuerzburg, Wuerzburg, Germany; h Department of Medical Informatics, University Medical Center Göttingen, Göttingen, Germany; i Department of Paediatrics, University Hospital Wuerzburg, Wuerzburg, Germany; j University Hospital Würzburg, Department of Anaesthesiology, Intensive Care, Emergency and Pain Medicine, Würzburg, Germany; k Synergy Research and Experimental Medicine Lab, Medical Clinic III, University Hospital Bonn, Bonn, Germany; l Staff Unit for Scientific & Medical Technology Development & Coordination (MWTek), Commercial Directorate, University Hospital Bonn, Bonn, Germany; m Applied Medical Informatics (AMI) Lab, Institute for Medical Biometry, Informatics & Epidemiology (IMBIE), University Hospital Bonn, Bonn, Germany; n Applied Mathematical Physiology (AMP) Lab, Department of Anesthesiology and Intensive Care Medicine, University Hospital Bonn, Bonn, Germany

**Keywords:** Incidental SARS-CoV-2, COVID-19, Hospitalization rate

## Abstract

**Purpose:**

Until now, the Hospitalization Rate (HR) served as an indicator (among others) for the COVID-19 associated healthcare burden. To ensure that the HR accomplishes its full potential, hospitalizations caused by COVID-19 (primary cases) and hospitalizations of patients with incidental positive SARS-CoV-2 test results (incidental cases) must be differentiated. The aim of this study was to synthesize the existing evidence on differentiation criteria between hospitalizations of primary cases and incidental cases.

**Methods:**

An online survey of the members of the German Network University Medicine (NUM) was conducted. Additionally, senior clinicians with expertise in COVID-19 care were invited for qualitative, semi-structured interviews. Furthermore, a rapid literature review was undertaken on publications between 03/2020 and 12/2022.

**Results:**

In the online survey (n=30, response rate 56%), pneumonia and acute upper respiratory tract infections were the most indicative diagnoses for a primary case. In contrast, malignant neoplasms and acute myocardial infarctions were most likely to be associated with incidental cases. According to the experts (n=6), the diagnosis, ward, and type of admission (emergency or elective), low oxygen saturation, need for supplemental oxygen, and initiation of COVID-19 therapy point to a primary case. The literature review found that respiratory syndromes and symptoms, oxygen support, and elevated levels of inflammatory markers were associated with primary cases.

**Conclusion:**

There are parameters for the differentiation of primary from incidental cases to improve the objective of the HR. Ultimately, an updated HR has the potential to serve as a more accurate indicator of the COVID-19 associated healthcare burden.

## Introduction

Severe Acute Respiratory Syndrome coronavirus 2 (SARS-CoV-2), causing the Coronavirus Disease 2019 (COVID-19), was first reported in the China's Wuhan province in January 2020. Shortly afterwards, the World Health Organisation (WHO) declared it to be a virus of international concern and as of 21 January 2024, the WHO has reported 7,023,271 deaths associated with the virus worldwide [1].

The mode of transmission of SARS-CoV-2 is contact-free i.e., via the generation and spread of respiratory particles. Transmission can take place within short and long distances, the respiratory particles can be introduced through mouth, nose, or eyes of the susceptible person [2]. COVID-19 can manifest in many clinical forms, from common flu symptoms such as cough and myalgia to acute respiratory failure requiring mechanical ventilation and circulatory support in the form of extracorporeal membrane oxygenation.

In the course of the SARS-CoV-2 pandemic several parameters have been used to inform the population and guide policies to (de-)escalate local infection control and Public Health measures. In the first year of the pandemic, the regional seven-day incidence (per 100,000 inhabitants) was used as one of the main guidance parameters, both locally and world-wide.^.^ Over time this indicator became less precise for the true healthcare-associated burden and in Germany it evolved into an indicator set. This set additionally included the seven-day incidence of new infections differentiated by age, the COVID-19-associated hospitalization rate (HR), and the percentage of people undergoing intensive care treatment [3]. These were arguably appropriate indicators, however, each single indicator covers a different aspect of the health care burden [4,5]. The reporting of the HR relied, therefore, on the admitted patients' positive for SARS-CoV-2 in the last seven days per 100.000 inhabitants, driven by the assumption that every SARS-CoV-2 positive patient has COVID-19.

However, with the infection- and vaccine-induced immunity and the appearance of lower virulence variants such as those in the Omicron lineages, the HR overestimated the number of hospitalized patients [6]. Partly due to misclassified COVID-19 patients, and because it neither considers the asymptomatic patients hospitalized with an incidentally positive SARS-CoV-2 test, nor the mildly diseased patients with clinically irrelevant COVID-19 that were admitted with a different diagnosis. Maintenance of SARS-CoV-2 screening strategies upon hospital admission as well as the increased community prevalence, also influenced the HR and led to potential over-reporting. In addition, residual RT-PCR positivity after convalescence is not considered, even though positive results may persist up to two months after the initial infection [7,8]. Under these circumstances, the current HR does not mirror and stratify the two mentioned stances of the circulating SARS-CoV-2 variant.

Therefore, a redefined HR that can differentiate between patients hospitalized due to COVID-19 (primary cases) and patients incidentally testing positive for SARS-CoV-2 at admission (incidental cases) is urgently needed. It should contain routine, easily accessible, and transferable parameters that allow (semi-)automatic surveillance without adding workload. This approach should enable in best case real-time reporting and help to anticipate the onset of new pandemic waves with new virus (sub) lineages with notably higher Public Health concerns. It could also facilitate a more differentiated approach to both public health interventions and hospital planning i.e., primary cases might be in need for different treatment regimes than incidental cases, influencing resource planning on a hospital level. Public health interventions such as opening or closing of places or the need to wear masks is also informed by disease severity and could be mis-informed by too-high hospitalization numbers without the differentiation.

To enhance the potential of the current HR, documentation practices and care procedures must be considered as well as the existing evidence from publications.

The aim of this paper is to summarize the best possible evidence on how to differentiate hospital admissions due to COVID-19 primary cases from hospitalizations with an incidental SARS-CoV-2 infection. We combined evidence from a rapid systematic literature review, as well as evidence from a structured online survey and semi-structured qualitative expert interviews. The results aim to support the development of predictive models using data via easy assessment of patient's electronic health records [9].

## Methods

This research is part of the *Network University Medicine* (NUM; University Medicine National Research Network on Covid-19) project *CODEX+* (COVID-19 Collaborative Data Exchange and Usage). This evidence synthesis is part of a larger study using an exploratory sequential mixed methods design [10]. The synthesis includes (a) an online survey, (b) semi-structured qualitative expert interviews and (c) a systematic rapid review of the literature. The online survey was conducted to get insight from hospital physicians including their personal experiences in factors associated with primary cases or incidental cases. The expert interviews were conducted to get a more thorough knowledge, also distinguishing between practices and distinctions between different wards. The review was conducted to get a broad sense of the evidence collected in the literature and also to externally rate our results.

a. Online survey

First, an online survey was conducted among the members of the German Network University Medicine (NUM) which includes a total of 36 German Academic Medical Centres. The NUM Network was established in April 2021 by the German Ministry of Education and Research with the overarching aim to establish a network of all German university hospitals to coordinate pandemic research [11].

The survey was conducted as an anonymous online survey on the platform SoSciSurvey [12]. It was distributed to physicians working with COVID-19 patients through the local NUM coordination units. The online survey consisted of closed-ended and open-ended questions about the typical presentation of primary and incidental cases at admission to hospital, typical data that are observed upon admission or shortly after, and the local availability of these data. Lists of potentially indicative diagnoses, therapies, symptoms and clinical parameters were presented in the following way: “Should the following parameters be given as an indication of whether a person has been admitted as a primary case or whether the infection was more likely to be incidental?” Parameters mentioned included e.g. admission diagnoses, admission to ICU, age, or number of vaccinations. For each potential indicator, the experts voted yes or no. They had also the opportunity to add free text. In a second step, respondents were asked to indicate the degree of decision relevance for each indicator (very likely, likely, possible, and very unlikely). Also, they were asked if documenting this indicator could be considered as easy or complicated, serving as information if that indicator could easily transferred into a point-of-care prediction model to correctly identify a patient as primary or incidental.

b. Semi-structured Qualitative interviews

To elicit the practical experience of the treating physicians, we conducted in-depth, semi-structured qualitative interviews based on first results from the online survey.

German and international experts from the field of intensive care medicine, paediatrics, emergency medicine and internal medicine were purposively recruited from the authors' professional network, the local NUM task forces and from respondents of the online survey who had agreed to be re-contacted. We included respondents if they were working at a tertiary academic medical centre. While most interview partners work in the German hospital context, we also contacted international experts to broaden our understanding of care delivered to patients with SARS-CoV-2 outside of the German hospital setting.

All potential participants were informed of data protection measures via an Informed Consent Form (ICF) that was sent to them before the interview. Interviews were held through an online video conference and audio recorded. The data extraction was done anonymously.

We conducted semi-structured interviews following a self-developed interview guideline covering the following topics: clinical experience and personal background, typical signs and symptoms, clinical parameters, and therapy options in patients with COVID-19.

Interviews were conducted by researchers with experience in qualitative research. A minimum of two researchers were present at each interview to ensure that assumptions and mentioning of new themes could be constantly discussed.

Information was extracted from the audio files consecutively after the interview using a Microsoft Access database. The results were analysed descriptively. Parameters were summarized where possible into four broad categories: characteristics associated to a primary case, characteristics associated to an incident case, characteristics differentiating primary from incidental cases, and characteristics which made the differentiation more complex.

c. Rapid systematic literature review

The review followed the Preferred Reporting Items for Systematic Reviews and Meta-Analyses (PRISMA) [13] and was registered with PROSPERO (CRD42022376761). In accordance with the systematic approach of a rapid review [[14], [15], [16]], a literature search was conducted in international medical literature databases using two separate search strings at the two involved research sites to have a broader and more comprehensive approach. Search strings and review steps can be found in Table II, Table III/Appendix. The search was limited to the period from March 1^st^, 2020, until August 15^th^, 2022 and English and German papers.

The final query was performed on August 24^th^, 2022 and was subsequently completed by hand search up to December 18^th^, 2022.

In the first phase, we applied the inclusion and exclusion criteria for abstract screening. Abstracts and full-texts were screened by two reviewers independently using the exclusion/inclusion criteria. Two other reviewers were consulted in case of disagreement and a decision was reached after discussion. The next phase included full-text screening and data extraction. In the final phase we checked papers for eligibility to answer the research question. We included all studies that reported characteristics of hospitalized (≥1 night) patients with confirmed SARS-CoV-2 infection without restriction for patient group, age, or sex. We restricted study settings to hospitals. We included studies regardless of publication status (see Table 3/Appendix).

Studies were ineligible if they did not report differentiation characteristics that were associated with primary and incidental cases, if they focused on a specific therapy, or a different illness or if they did not withstand the prior determined criteria for abstracts in the full texts. The ineligible studies were then excluded from the data synthesis.

We extracted information on country/region, setting, study type, number of included patients, number of primary incidental cases, type of testing, age-group, gender, as well as the inclusion of children using a Microsoft Access database. Potential characteristics were stratified by diagnoses, symptoms, clinical features, and laboratory parameters. Each paper was read and extracted by two reviewers. In case of discordance, at least one more reviewer was included. A formal risk of bias assessment was not conducted [[14], [15], [16]]. Quality assessment was conducted by two authors using the Critical Appraisal Skills Programme (CASP) tool to assess methodological limitations in a systematic review [17].

## Results

### Online survey

The survey was sent out to 53 experts through the NUM local coordinating units, yielding a response rate of 56% (30/53). Respondents were predominantly specialists in internal medicine (13), emergency/intensive care (11), infectious diseases or infection prevention and control (5), and paediatrics (5). All respondents had at least five years of clinical experience in their respective fields.

Of all respondents, 90% named acute pneumonia and acute upper respiratory infections as the most likely diagnoses to define a primary case (in combination with a positive SARS-CoV-2 test).

Respondents reported several diagnoses that were likely indicative for an incidental case, among these were malignant neoplasms (33%), acute myocardial infarction (33%) and its clinical form ST-elevation myocardial infarction (STEMI; 37%).

Additionally, age, gender, frequency of SARS-CoV-2 vaccinations, recovery status and time since recovery, admission via emergency department, need for oxygen supplementation, ECMO therapy, need for corticosteroid treatment, viral load or cycle threshold value, blood pressure, C-reactive protein, procalcitonin, oxygen saturation, invasive or non-invasive ventilation, admission to intensive care unit and the comorbidities such as lung diseases, heart diseases, obesity/overweight, kidney diseases and blood pressure (systolic and diastolic) were reported as relevant by more than 50% of the participants (Table 1, Appendix).

### Expert interviews

A total of six interviews were conducted between September and November 2022. Interview length ranged from 15 to 28 minutes.

Experts were senior physicians in their respective field and were all involved in the care of COVID-19 patients throughout the pandemic. They were working in tertiary care hospitals in the fields of pneumology, intensive care, paediatrics, and emergency care. Four experts from Germany, one from Austria and one from Slovenia took part in the interviews.

A low oxygen saturation and increased C-reactive protein (CRP) levels were mentioned as typical characteristics for primary cases. Patients presented with cough and high temperature, a combination of headache and sore throat, with respiratory failure, and/or with a need for respiratory support. Smell and taste disturbances and gastrointestinal symptoms were commonly reported symptoms during the Delta wave. Symptoms typical for newborns and young children were croup-like cough, decreased fluid intake, and febrile convulsion. Comorbidities more often associated with primary cases were immunosuppression, asthma, diabetes, and obesity. For patients that required imaging, infiltrates were associated with primary cases. Additionally, patients with primary COVID-19 were likely to be admitted through emergency services, with fewer tests and diagnoses (only the most necessary). Children were less likely to be admitted because of severe disease progression but rather for general precaution or parental insistence.

Experts characterized incidental cases by normal oxygen saturation, inconclusive imaging, indicative test results of other infections (e.g., RSV, urinary tract infections), or hyperinflammation syndrome. Pre-existing conditions such as chronic neurological or neuromuscular conditions, COPD with worsening symptoms, severe heart defects in children, or severe multiple disability in children, were also associated with a hospitalization as an incidental case.

Experts characterized incidental cases as cases without a “typical” presentation. Disease progression might be an indicator because primary COVID-19 cases evolved slower to a critical state than other viral infections. Comorbidities were described as a main challenge. To give an example, patients presenting with cardiovascular comorbidities and a positive SARS-CoV-2 test might have several problems necessitating hospitalization that could be attributed to both the comorbidity and the SARS-CoV-2 infection.

### Rapid systematic literature review

**Figure 1:**
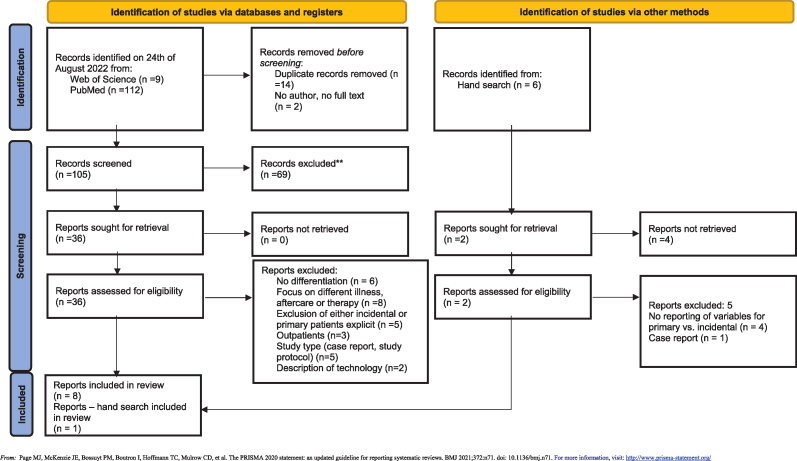
Flowchart.

A total of 121 publications were identified (PubMed 112, Web of Science 9), with 14 duplicates. After reviewing the abstracts 36 articles were considered relevant for full text screening. Twelve articles were included in the end for appraisal. We found six additional articles by manual searching, and two of those were included for appraisal. Finally, eight papers were included in the analysis (Figure 1/Appendix).

The articles included originated from 5 countries (USA, Canada, Costa Rica, Iran, and the Netherlands) with a total of 4085 patients. All studies reported differentiation criteria between primary and incidental cases. Of the eight selected publications, seven reported information about children, and five of those reported the characteristics of each of the predefined outcomes separately.

All extracted parameters are reported in Table 4/Appendix. The summarized results are depicted in Table 2.

The predominant diagnoses among primary cases included acute respiratory or cardiovascular decompensation diagnoses and gastrointestinal and genitourinary tract diagnoses (some of which were chronic conditions (e.g., Crohn's disease)). Other acute and sudden onset diagnoses like TIA, sepsis, DKA and subdural hematoma were also outlined. Most of the symptoms related to primary cases were gastrointestinal and respiratory, accompanied by symptoms generally associated with infection or inflammation. Of the clinical features, respiratory failure and the need for oxygen support along with treatment in an ICU indicated a primary case. Laboratory parameters reported for primary cases included blood count abnormalities and elevated levels of inflammatory markers. In addition, paediatric primary cases had diagnoses and symptoms of gastrointestinal nature. COVID-19-associated coagulopathy was also observed in this group.

Among incidental cases, there was a wide range of diagnoses with little specificity, except for trauma which was indicative of incidental cases. No specific laboratory parameters or symptoms were associated with incidental cases.

## Discussion

The proportion of patients admitted to the hospital due to COVID-19 remains an important indicator of the burden of disease and the burden of the pandemic in health care systems. However, in a situation with a high background prevalence of infection, differentiating primary cases from incidental SARS-CoV-2 infections remains difficult. Several guidelines provide stratification information for the severity of COVID-19 disease [3,18,19] and a number of research articles identify incidental patients as misclassified COVID-19 patients [4,5] but evidence for differentiating between primary vs. incidental upon admission is scarce.

### Indicators potentially identifying primary cases

One main factor identified as potentially indicative by literature and expert opinion was oxygen saturation or the need for oxygen therapy. This parameter has clear benefits, it is easy to measure and verify and it is already one of the key indicators of the severity of the disease or mortality in studies and guidelines [3,18,20,21]. This is in line with the evidence found in our review, in which low oxygen saturation or the need for oxygen was an indicator for primary cases [[22], [23], [24], [25]]. To use oxygen saturation as an indicator, hospitals need to document saturation continuously, preferably automatically. In our survey, 50% of the participating German experts rated the effort to document this data as low and indicated that this parameter as helpful.

A potentially valuable indicator could be the admission diagnosis. The first diagnosis made after emergency admission is based on symptoms and a first clinical evaluation. Research on the consistency of emergency admission diagnoses and primary diagnoses reports a wide range, with a lowest match for sepsis and the highest for appendicitis or concussion (>90%); most range around a 50% match of both diagnoses [26,27]. Using the admission diagnosis as the only indicator for indicator might therefore not be sufficient to identify primary cases. For some emergency cases (i.e., acute trauma), it might, however, have high face validity to identify an incidental case.

The main diagnoses associated with primary cases in our review did not differentiate well. Several diagnoses were uncertain in differentiating between primary or incidental cases, such as preterm labour or immune dysfunction [23]. However, some diagnoses were indicative for incidental cases, including STEMI, fractures, appendicitis, brain tumour, or elective surgical procedures [5,23,28,29]. Several of these diagnoses were identified in the online survey as being associated to an incidental case (see Table 1).

**Table I:** Results of the expert online survey.

	Very likely/likely	possible	Exclusion
For which of the following COVID-19 **main admission diagnoses**, in combination with a positive SARS-CoV-2 PCR detection, is it certain/probable/possible that the reason for admission as a primary case?			
COVID-19, virus identified (U07.1)	14 (47%)	15 (50%)	
COVID-19, virus not identified (U07.2)	1 (3%)	24 (80%)	
Personal history of COVID-19 (U08.9)	2 (7%)	26 (87%)	
Multisystem inflammatory syndrome associated with COVID-19, unspecified (U10.9)	19 (63%)	8 (27%)	
COVID-19 with pneumonia (J12.8 - pneumonia caused by other viruses)	27 (90%)	2 (7%)	
In which of the following additional **main admission diagnoses**, in combination with a positive SARS-CoV-2 PCR test, is it certain/probable/possible/excluded that this is a primary case (Definition of exclusion: If one of the diagnoses is present, admission as a primary case can be excluded.)			
Malignant neoplasms (C00-C97)	0 (0%)	17 (57%)	10 (33%)
Transient ischemic attack (TIA) (G45)	0 (0%)	20 (67%)	5 (17%)
Postviral fatigue syndrome (G93.3)	1 (3%)	24 (80%)	2 (7%)
Acute myocardial infarction (I21)	0 (0%)	15 (50%)	10 (33%)
ST elevation myocardial infarction (STEMI)	0 (0%)	14 (47%)	11 (37%)
Non-ST elevation myocardial infarction (NSTEMI)	0 (0%)	16 (53%)	9 (30%)
Pulmonary embolism (I26)	3 (10%)	20 (67%)	2 (7%)
Stroke, stroke (I64)	0 (0%)	20 (67%)	6 (20%)
Acute upper respiratory tract infection (J06)	22 (73%)	6 (20%)	0 (0%)
Acute pneumonia (J18)	22 (73%)	7 (23%)	0 (0%)
Adult respiratory distress syndrome [ARDS] (J80)	21 (70%)	6 (20%)	1 (3%)
Damage to newborn from maternal COVID-19 infection (P00.2)	10 (33%)	8 (27%)	0 (0%)
Heartbeat disorders (R00)	1 (3%)	19 (63%)	5 (17%)
Cough (R05)	16 (53%)	12 (40%)	1 (3%)
Dyspnea (R06.0)	14 (47%)	15 (50%)	0 (0%)
Disturbances of smell and taste (R43)	19 (63%)	8 (27%)	1 (3%)
Other specified fever (R50.88)	16 (53%)	12 (40%)	0 (0%)
Septic shock (R57.2)	5 (17%)	17 (57%)	6 (20%)
Systemic inflammatory response syndrome [SIRS] (R65)	10 (33%)	15 (50%)	3 (10%)
Multiple organ failure (R68.8)	9 (30%)	16 (53%)	3 (10%)
Isolation as a prophylactic measure for COVID-19 (SARS-CoV-2) (Z29.0)	12 (40%)	15 (50%)	0 (0%)
Undesirable side effects of using COVID-19 vaccines (U12.9)	0 (0%)	20 (67%)	7 (23%)
Other mental disorders due to brain damage or dysfunction or physical illness (F06)	0 (0%)	19 (63%)	6 (20%)
Diseases of the liver (K70-K77)	1 (3%)	16 (53%)	9 (30%)
Diseases of the arteries, arterioles, and capillaries (I70-I79)	0 (0%)	17 (57%)	8 (27%)
Organic, including symptomatic, mental disorders (F00-F09)	0 (0%)	17 (57%)	7 (23%)
Cerebral palsy and other paralytic syndromes (G80-G83)	0 (0%)	16 (53%)	9 (30%)

**Table II:** Basic information on the included publications.

First Author	Pub. Year	Study type	Total Patientsincluded	Femalen (%)	IncidentalCases	PrimaryCases	Age (years)	Type of test
Klann, J. G. et al.[23]	2022	Cross-sectional	1123	371 (48%)	359	764	All	PCR
Drouin, O.[29]	2021	Cohort	264	122 (47)	150	100	<18	N/A
Tehseen, Sarah I et al.[30]	2022	Cross-sectional	985	354 (38)	242	673	<18	N/A
Tsai, J. et al.[5]	2021	Cross-sectional	346	159 (46)	43	303	All	N/A
Voor In 't Holt, Anne F et al.[32]	2022	Cross-sectional	172	78 (45)	54	72	0-90	PCR
Wanga, Valentine et al. [22]	2021	Cross-sectional	915	437(48)	177	738	<18	Antigen test /PCR/Antibody test
Webb, N.E; Osburn T.S.[25]	2021	Case-series	163	69 (42)	58	88	<22	Antigen test/PCR
Kushner et al.[19]	2021	Case-series	117	58 (50)	46	71	1.5-14	PCR

Note: PCR= polymerase chain reaction, N/A=not available

**Table III:** a and b: Reported characteristics associated with patients admitted to hospital because of COVID-19 (primary cases, III.a) and those associated with patients admitted to hospital with a SARS-CoV-2 infection (incidental cases, IIIb).

a. Reported characteristics associated with primary cases									
Variables∖Paper		Klann J. G.	Drouin O.	Tsai J. et al.	Voor In ‘t Holt, Anne F.	Webb, N.E.	Kushner	Wanga V.	Tehseen Sarah I.
Diagnoses	Respiratory	x	x	x	-	x	x	-	-
	Cardiovascular	x	x	x	-	-	-	-	-
	Gastrointestinal	x	x	x	-	-	-	-	-
	Urogenital	x	-	x	-	-	-	-	-
Symptoms	Respiratory	-	x	x	-	x	-	-	-
	Cardiovascular	-	-	x	-	-	-	-	x
	Gastrointestinal	-	x	x	-	x	-	-	-
	Urogenital	-	-	-	-	-	-	-	-
	Infection/Inflammation	-	x	-	-	x	-	-	-
Clinical features	Respiratory	x	-	-	-	x	-	-	x
	Cardiovascular	x	-	-	-	-	-	-	-
	Comorbidities	-	-	-	x	x	x	x	-
	Oxygen support	-	-	-	x	x	-	x	-
	ICU admission	-	-	-	-	x	-	x	x
Laboratory parameters	Cardiovascular	x	x	x	-	-	-	-	-
	Inflammatory	x	x	x	-	-	-	-	-
	Gastrointestinal	x	-	x	-	-	-	-	-

**b. Reported characteristics associated with** with an incidental SARS-CoV-2 infection									
Variables∖Paper		Klann J. G. et al.	Drouin O.	Tsai J. et al.	Voor In 't Holt A. F. et al.	Webb N. E., Osburn T. S.	Kushner et al.		
Diagnoses	Traumatic injury	x	-	x	-	x	-		
	Infectious disease (otherthan SARS-CoV-2/COVID-19)	x	-	-	-	-	x		
	Cardiovascular /Cerebrovascular	-	-	-	-	-	x		
	Gastrointestinal	-	x	-	-	-	x		
	Urogenital	x	-	-	-	-	x		
	Other	x	x	x	-	-	x		
Symptoms	Respiratory	-	-	-	- ∗	-	-		
	Cardiovascular	-	-	-	-	-	-		
	Gastrointestinal	-	-	-	-	-	-		
	Urogenital	-	-	-	-	-	-		
	Infection/Inflammation	-	-	-	-	-	-		
Clinical features	Respiratory	-	-	-	-	-	-		
	Cardiovascular	-	-	-	-	-	-		
	Comorbidities	-	-	x	-	-∗	-		
	Oxygen support	-	-	-	-	-	-		
	ICU admission	-	-	-	-	-	-		
Laboratory parameters	Cardiovascular	-	-	-	-	-	-		
	Inflammatory	-	-	-	-	-	-		
	Gastrointestinal	-	-	-	-	-	-		

Both experts and literature identified clinical parameters and additional indicators associated specifically with primary or incidental cases. To identify primary cases, experts named recovery status and admission through emergency department. The clinical parameters associated with primary cases were the Ct value/viral load and CRP. Both were named repeatedly in expert interviews and were also found in the literature [23]. Admission through the emergency department is also an indicator found in both literature and interviews. The review yielded ICU admission (PICU admission for children) as an indicator associated with primary cases [22,25,30]. Specifically for children, the clinical presentation of COVID-19 seems to have predominantly gastro-intestinal and neurological elements and depends on the current circulating variant [25,29].

Lymphopenia was named in one expert interview as associated with primary cases, and in one of the articles included in the review [29]. Especially for children, experts reported the importance to exclude other diagnoses, especially respiratory syncytial virus (RSV) infections, as the cause for hospitalization, since RSV infections would more likely lead to severe disease progression.

### Indicators potentially identifying incidental cases

Identification of clinical parameters associated with incidental cases is more challenging, as stated in an expert interview: “there is no typical incidental case” [translated from German to English]. Experts stated that the current circulating variant and their (sub)lineages are relevant, with potentially less virulent variants associated more with incidental cases. Several articles support this, stating a lower risk of hospitalization or severe disease progression with higher vaccination rates and during the Omicron wave [[31], [32], [33], [34], [35]]. Interestingly, only one of the included review articles reported vaccination as a criterion for differentiation [24]. However, the study had a small sample size and higher vaccination rates in primary cases. There is vast literature on the effect of vaccination status on risk of hospitalization or on severity of disease [[36], [37], [38], [39], [40]], but no study used this indicator to differentiate in our review. Especially in children, relying on symptoms might not be feasible since many infections are asymptomatic at young ages [41]. One possible explanation for this could be that vaccination status is not consistently recorded upon admission and is dependent on the patient's willingness to disclose such information.

### Strengths and limitations

The strength of our study is the combination of mixed methods to identify the current evidence on the definition of incidental and primary hospitalizations. Therefore, we could include international evidence and the experience of current care practice in hospitals of different disciplines.

However, some limitations must be considered: The expert survey and interviews only included a small number of physicians from tertiary care hospitals treating a selected population of patients. We chose this group due to its strong clinical background and knowledge of the treatment of COVID-19 patients. While our response rate for the survey can be considered good, at 56%, we only invited a total of 53 experts. We did this to ensure the high level of expertise of invited experts. Since the answers are internally consistent and are also in line with the information we gathered in the interviews, we do not think that the lower number of respondents lead to wrong assumptions. In the systematic rapid review, we screened only German and English articles and followed the methodology of a rapid review. While this approach was chosen to allow a timely investigation, a full structured systematic literature review might yield more thorough results.

## Conclusions

The results of this study can be used to refine the hospitalization rate and restore the potential to serve as an accurate indicator of the COVID-19 associated healthcare burden. While incidental cases will also increase burden for the hospital in terms of isolation and protective measures for staff, a differentiation of primary and incidental cases will improve prediction of acute and intensive care capacities. Additionally, this differentiation will aid in defining disease severity and, consequently, improving the overall treatment of patients. Also, regional and national reporting of hospitalization rate can improve the extent of public health measures in the general population, if a clear distinction between primary and incidental cases can be made. While achieving perfect prediction is challenging given the complex nature of the disease and its associated symptoms and signs, enhancements in prediction accuracy can certainly be achieved by incorporating additional information.

Our synthesis of evidence from expert opinion and literature is the first to reveal a number of parameters that might facilitate the differentiation between primary and incidental COVID-19 cases. We identified potential parameters to improve the meaning of the HR. The evidence for incidental cases varied widely, but for the primary cases we could identify common parameters. Our results can inform a predictive modelling that might improve the HR as a more comprehensive tool for the pandemic surveillance, irrespective of virus variants and also adaptable for potential current and future pandemics as well as for the surveillance of endemic and epidemic infections. The implementation of such an updated HR can facilitate the prediction of healthcare-associated burden in a pandemic, including economic questions or health-related consequences. In a next step, the identified indicators were tested in a prediction model, concluding in two algorithms that could be used as a precise surveillance tool to support pandemic preparedness [7].

To summarise, the results presented in this study can be helpful for pandemic preparedness in terms of facilitating hospital and patient management, improving policy advice and increasing the quality of guidance parameters for public communication and information.

## Author contributions

Conceptualization: EG, MM, DK, RS, SS; Methodology: AMK, EG, DK, SS, SB, MM, RS, MB; Formal analysis and investigation: MM, DK, MB, AMK, RS; Writing - original draft preparation: SS, EG, DK, MM, AMK, MB, SB; Writing - review and editing: All Authors; Funding acquisition: PM, SB, DK, SZ, SS, EG, 10.13039/501100014710PB, RB, MK, MM; Resources: All authors; Supervision: EG, SS.

## Conflict of interests

There are no conflict of interests declared by the authors. The authors have no relevant financial or non-financial interests to disclose.

## Funding statement

The CODEX+ project of the National University Medicine Research Network was funded by the 10.13039/501100002347Federal Ministry of Education and Research of Germany (Bundesministerium fuer Bildung und Forschung (10.13039/501100002347BMBF)) grant number 01KX2121.

## Ethics approval

The interviews were approved by the Ethics Committee and the Data Protection Officer of the Medical Faculty of the LMU Munich (22–0734).

The Ethics Committee of the Faculty of Medicine of the LMU Munich issued a waiver for the survey (22–0658 KB).

## Figures and Tables

**Table 1:** Online survey additional information.

	not relevant	relevant	n.a.
**Other criteria**			
Age	12 (40%)	16 (53%)	2 (7%)
Gender	24 (80%)	4 (13%)	2 (7%)
Number of SARS-CoV-2 vaccinations	13 (43%)	15 (50%)	2 (7%)
Type of SARS-CoV-2 vaccinations	18 (60%)	9 (30%)	3 (10%)
Recovery status	12 (40%)	18 (60%)	0 (0%)
Period from last SARS-CoV-2 infection	10 (33%)	20 (67%)	0 (0%)
**Health Services/treatments**			
Admission to intensive care unit	5 (17%)	21 (72%)	3 (10%)
Admission via emergency department	9 (31%)	17 (59%)	3 (10%)
ECMO therapy	5 (17%)	21 (72%)	3 (10%)
Invasive ventilation	4 (14%)	22 (76%)	3 (10%)
Non-invasive ventilation (e.g. NIV)	4 (14%)	22 (76%)	3 (10%)
Therapy with oxygen mask/nasal oxygen cannula	4 (14%)	22 (76%)	3 (10%)
Need for vasopressor therapy	10 (34%)	14 (48%)	5 (17%)
Need for therapy with corticosteroids	7 (24%)	18 (62%)	4 (14%)
**Measurements**			
Oxygen saturation	3 (11%)	23 (82%)	2 (7%)
Blood pressure (systolic and diastolic)	11 (39%)	15 (54%)	2 (7%)
Cycle threshold value (Ct value)	5 (18%)	22 (79%)	1 (4%)
Viral load	10 (36%)	15 (54%)	3 (11%)
D-dimers	11 (39%)	13 (46%)	4 (14%)
C-reactive protein (CRP)	10 (36%)	15 (54%)	3 (11%)
Procalcitonin	9 (32%)	16 (57%)	3 (11%)
N-terminal pro b-type natriuretic peptide (NT-proBNP)	12 (43%)	13 (46%)	3 (11%)
Cardio-specific troponin (cTn)	14 (50%)	11 (39%)	3 (11%)
Full blood count	12 (43%)	13 (46%)	3 (11%)
Differential count	12 (43%)	13 (46%)	3 (11%)
**Pre-existing conditions/comorbidities**			
Heart diseases	9 (31%)	17 (59%)	3 (10%)
Lung diseases	5 (17%)	21 (72%)	3 (10%)
Kidney diseases	10 (34%)	16 (55%)	3 (10%)
Cerebral circulatory disorders (e.g. stroke, TIA)	10 (34%)	14 (48%)	5 (17%)
Diabetes mellitus	12 (41%)	14 (48%)	3 (10%)
Obesity/overweight	9 (31%)	17 (59%)	3 (10%)
Peripheral Arterial Disease	13 (45%)	11 (38%)	5 (17%)
Gastrointestinal disease	11 (38%)	14 (48%)	4 (14%)
Dementia	13 (45%)	11 (38%)	5 (17%)
Hemiplegia/paralysis	16 (55%)	9 (31%)	4 (14%)
Malignancy/tumor	11 (38%)	14 (48%)	4 (14%)
AIDS/HIV	12 (41%)	13 (45%)	4 (14%)

**Table 2:** The search string used in the literature search.

Segment (German)	Search Term (English)	Link
Hauptbefundlich	Primary	AND
Nebenbefundlich	Incidental	AND
SARS-CoV-2	SARS-CoV-2 OR COVID-19 OR coronavirus OR severe acute respiratory syndrome	AND
Hospitalisierung	Hospitalisation OR hospitalization OR hospitalised OR hospitalized OR hospital OR inpatient OR patient	AND
Charakteristik	Characteristic OR diagnosis OR diagnosed OR symptom OR symptomatic OR difference OR differentiating OR attribute OR feature	AND

**Table 3:** Inclusion and exclusion criteria for the rapid review.

Criteria for inclusion	Criteria for exclusion
German or English	Other language
All patient groups, age groups, sex/genders	Animal studies
Confirmed SARS-CoV-2 infection	Patients with an unconfirmed or suspected infection that could not be verified
Hospitalized patients (≥ 1 night), context acute care hospital, all departments	Outpatient clinics, other medical non-hospital, non-acute care facilities like care homes, rehabilitation facilities
All study types excluding case reports	Case reports
Period of observation 03/01/2020 – 08/15/2022	Period of observation before the COVID-19 pandemic

**Table 4:** Characteristics of patients admitted to hospital because of COVID-19 (primary cases) and with SARS-CoV-2 infection (incidental cases).

First Author		Diagnoses	Symptoms/Signs	Clinical Features	Laboratory parameters
**Primary cases**					
Klann, J. G. et al.[23]	Respiratory:	COVID-19, virus identified, Other Viral Pneumonia, Acute Respiratory Failure with Hypoxia, Hypoxemia, Pneumonia due to COVID-19	–	Respiratory insufficiency	–
	Cardiovascular:	Essential (primary) hypertension	–	Blood clots, Hemodynamic changes	Cardiac Troponin, INR, PTT
	Gastrointestinal:	Gastro-esophageal reflux disease without esophagitis	–	–	Total Bilirubin
	Urogenital:	Acute kidney failure	–	–	–
	Other:	Hyperlipidemia, Personal history of nicotine dependence	–	Other symptoms for COVID-19 (not defined)	CRP, Ferritin, LDH, Procalcitonin, Lymphocyte count, Neutrophil count
Tsai, J. et al.[5]	Respiratory:	Pneumonia, ARDS, Bronchitis, Chronic obstructive pulmonary disease exacerbation	Shortness of breath, Cough	–	–
	Cardiovascular:	Myocarditis, Management of immunosuppression status post heart transplant, Unstable angina, Hypertensive emergency	Chest pain	–	–
	Gastrointestinal:	Acute appendicitis, Acute cholecystitis, Acute gastrointestinal bleed, Crohn's disease, Food intolerance, Diverticulitis	Abdominal pain, Nausea, Vomiting	–	–
	Urogenital:	Urinary tract infection, Pelvic inflammatory disease, Pyelonephritis, Acute kidney injury, Vaginal bleeding	–	–	–
	Other:	Metabolic encephalopathy, Questionable subdural hematoma, Paresthesia, Bell's palsy, Transient ischemic attack, Sepsis, DKA, Uncontrolled diabetes, Osteomyelitis, *Escherichia coli* infection, Delivery of preterm infant, Postoperative wound complication, L4-5 stenosis	Fever, Syncope, Joint pain and swelling	Weight loss, Dehydration	Hyponatremia
Voor In 't Holt, Anne F et al. [32]	Respiratory:	–	–	Oxygen therapy on admission	–
	Cardiovascular:	–	–	–	–
	Gastrointestinal:	–	–	–	–
	Urogenital:	–	–	–	–
	Other:	–	–	Solid organ transplantation, COVID-19 Vaccination	–
Wanga, Valentine et al. [22]	Respiratory:	–	–	Oxygen support, RSV coinfection	–
	Cardiovascular:	–	–	–	–
	Gastrointestinal:	–	–	–	–
	Urogenital:	–	–	–	–
	Other:	–	–	Obesity, ICU admission	–
Tehseen et al. [30]	Respiratory:	–	–	Respiratory support	–
	Cardiovascular:	–	Hemorrhage, Thrombosis	–	–
	Gastrointestinal:	–	–	–	–
	Urogenital:	–	–	–	–
	Other:	–	–	ICU admission, Anticoagulation	–
Drouin, O. et al. [29]	Respiratory:	Pneumonia, Bronchiolitis, ARDS. Comorbidity: , Chronic lung disease (Asthma)	Cough, Runny nose, Sneezing, Loss of smell	–	–
	Cardiovascular:	Hypotension, Coagulopathy, Cardiac dysfunction (not specified)	–	–	Thrombocytosis
	Gastrointestinal:	Hepatitis	Sore throat, Vomiting, Loss of appetite, Diarrhea, Loss of taste	–	–
	Urogenital:	–	–	–	–
	Other:	Seizures, Encephalopathy, Cytokine storm/MAS, Comorbidities: Chronic encephalopathy with severe neurodisability, Obesity, Epilepsy, Neurodevelopmental comorbidity (cerebral palsy)	Fever, Headache, Lethargy, Rash, Conjunctivitis, Muscle aches,	–	Lymphopenia, Anemia, Neutropenia, Cytokine storm (not defined)
Webb, N.E; Osburn T.S. [25]	Respiratory:	Asthma	Respiratory symptoms (not specified)	Hypoxia, Supplemental oxygen therapy, Duration of oxygen therapy (>48h), invasive respiratory support	–
	Cardiovascular:	–	–	–	–
	Gastrointestinal:	–	GI symptoms (not specified)	–	–
	Urogenital:	–	–	–	–
	Other:	DKA, Obesity	Fever	PICU Admission, Dexamethasone administration, Hospital average LOS	–
Kushner et al.[19]	Respiratory:	Respiratory viral infection	–	–	–
	Cardiovascular:	–	–	–	–
	Gastrointestinal:	–	–	–	–
	Urogenital:	–	–	–	–
	Other:	Worsening seizure, BRUE	–	–	–
**Incidental cases**					
Klann, J. G. et al. [23]	Traumatic injury:	Trauma (not specified)	–	–	–
	Infectious:	COVID-19 virus identified, Other viral pneumonia	–	–	–
	Cerebral-/Cardiovascular:	–	–	–	–
	Gastrointestinal:	–	–	–	–
	Urogenital:	Acute kidney failure, End-stage renal disease	–	–	–
	Other:	Type 2 DM with diabetic chronic kidney disease, Type 2 DM without complications, Anemia, Acidosis, Acute respiratory failure with hypoxia, Full-term labor; Thrombocytopenia	–	Other than COVID-19 (not specified)	–
Drouin, O. [29]	Traumatic injury:	–	–	–	–
	Infectious:	–	–	–	–
	Cerebral-/Cardiovascular:	–	–	–	–
	Gastrointestinal:	IBD	–	–	–
	Urogenital:	–	–	–	–
	Other:	Bone marrow transplantation, Psychiatric conditions, Prematurely born.	–	–	–
Tsai, J. et al. [5]	Traumatic injury:	Traumatic or accidental-related injury (not specified)	–	–	–
	Infectious:	–	–	–	–
	Cerebral-/Cardiovascular:	–	–	–	–
	Gastrointestinal:	–	–	–	–
	Urogenital:	–	–	Hemodialysis	–
	Other:	Substance use/ psychiatric illness, Full-term delivery, Elective surgical procedure, Osteosarcoma, Cancer metastasis to bone	–	–	–
Voor In 't Holt, Anne F et al. [32]		COVID-19 is not the cause of hospitalization (not specified)	Non- or only mild COVID-19 symptoms (not specified)	No medical treatment for COVID-19 symptoms (not specified)	
Webb, N.E; Osburn T.S. [25]		Skeletal fracture		Absence of comorbidities	
Kushner et al. [19]	Traumatic injury:	–	–	–	–
	Infectious:	Odontogenic infection, Pyelonephritis, Cellulitis, Central line infection, Neonatal sepsis with *E. coli*, Septic joint, Tubo-ovarial abscess, Urinary tract infection, *S. enterica* gastroenteritis, Bacterial lymphadenitis	–	–	–
	Cerebral-/Cardiovascular:	Moya Moya, Seizure with underlying neurologic abnormalities, Cardiac surgery or catheterization, Central line placement, Palpitations, AVM, Lymphangioma	–	–	–
	Gastrointestinal:	Enteric tube placement or replacement, Acute appendicitis, Intentional ingestion, Acute on chronic liver failure, IBD	–	–	–
	Urogenital:	Nephrectomy and dialysis catheter placement, complication, Nephrolithiasis and urinary retention, Abnormal uterine bleeding	–	–	–
	Other:	Transplant surgery, Chemotherapy admission, Pancytopenia in liver transplant on myelosuppressive medication, Severe acute on chronic anemia., DKA, Anaphylaxis from ingestion of food allergen, Spinal cord mass, Scoliosis surgery, Newborn delivered to SARS-CoV-2-positive mother, Lupus, Manic episode	–	–	–

Note: ARDS = acute respiratory distress syndrome, AST = aspartate aminotransferase, ALT = alanine aminotransferase, AVM = arteriovenous malformation, BRUE = brief resolved unexplained event, CVO = central vein occlusion, COHb = carboxyhemoglobin, CRP = C-reactive protein, CO = carbon monoxide, COPD= chronic obstructive pulmonary disease, DVT = deep vein thrombosis, DKA = diabetic ketoacidosis, DM = diabetes mellitus, FMF = familial mediterranean fever, GI = gastrointestinal, ICU = intensive care unit, IBD = inflammatory bowel disease, INR = international normalized ratio, LDH = lactate dehydrogenase, LOS = length of stay, MAS = macrophage activation syndrome, PTT = prothrombotic time, RSV = respiratory-syncytial virus, TIA = transient ischemic attack, VT = ventricular tachycardia. Marked with gray are papers reporting characteristics only for children.
